# A new extraction method of underglaze brown decorative pattern based on the coupling of single scale gamma correction and gray sharpening

**DOI:** 10.1371/journal.pone.0305118

**Published:** 2024-08-29

**Authors:** Tao Fang, Dashu Qin, Rumeng Zhang, Yu Jiang, Xue Rui

**Affiliations:** 1 Jingdezhen Ceramic University, Jingdezhen, Jiangxi Province, China; 2 Hangzhou City University, Hangzhou, Zhejiang Province, China; Hainan Normal University, CHINA

## Abstract

In order to solve the problem of image quality and morphological characteristics of primary underglaze brown decorative pattern extraction, this paper proposes a method of primary underglaze brown decorative pattern extraction based on the coupling of single scale gamma correction and gray sharpening. The single-scale gamma correction is combined with the gray sharpening method. The single-scale gamma correction improves the contrast and brightness of the image by nonlinear transformation, but may lead to the loss of image detail. Gray sharpening can enhance the high frequency component and improve the clarity of the image, but it will introduce noise. Combining these two technologies can compensate for their shortcomings. The experimental results show that this method can improve the efficiency of last element underglaze brown decorative pattern extraction by enhancing the image retention detail and reducing the influence of noise. The experimental results showed that F1Score, Miou(%), Recall, Precision and Accuracy(%) were 0.92745, 0.82253, 0.97942, 0.92458 and 0.92745, respectively.

## 1 Introduction

Ancient ceramics are an important part of the protection of China’s intangible cultural heritage. The restoration technology of ancient ceramics is an ancient and modern skill, aiming to repair the damaged ancient ceramics and make them return to their original appearance and artistic value. For a long time, although a large number of ceramic relics have been unearthed, the restoration and protection of ancient ceramics is not ideal due to history, professional knowledge and technical means. With the continuous innovation and application of science and technology, the ancient ceramic restoration and protection technology is also constantly innovating and developing. It is of great theoretical value and practical significance to explore the digital reproduction of ancient ceramic restoration and to promote the true restoration and permanent retention of ancient ceramic techniques and works.

Ming Dynasty underglaze brown decorative porcelain, produced in the Ming Dynasty, is one of the important representative works in the history of Chinese ceramics, with very high cultural and artistic value, and rarely passed down to modern times [1, 2]. At present, the spread of Ming Dynasty underglaze brown decorative is in a very dangerous situation, due to oxidation, pollution, corrosion and damage and other factors, resulting in damage and loss of its surface, which has an impact on its original beauty. Although China has applied many advanced technologies and methods in the protection of cultural relics, there are still some shortcomings [3–5].

First of all, some cultural relics protection technologies are not mature and perfect enough to achieve the optimal protection effect [6]. Secondly, some cultural relics protection units lack necessary equipment and technology, which makes it difficult to carry out cultural relics protection [7]. Therefore, in order to meet the needs of Ming Dynasty underglaze brown decorative protection, it is necessary to strengthen the research and innovation of cultural relic protection technology, and promote mature cultural relic protection technology and methods [8, 9]. With the introduction of digital technology and the evolution of people’s demand for the roles and functions of intangible cultural heritage cultural relics works, the technical concept of ancient ceramic restoration has undergone a great change. Some scholars, experts and even front-line restoration craftsmen all believe that the intervention of digital technology not only improves the efficiency and accuracy of traditional ancient ceramic restoration by hand, but also provides more rich and diversified means for the research and protection of ancient ceramics. Based on the digital protection of cultural relics, combined with the research of image enhancement and image segmentation, this paper proposes to automatically extract the patterns of underglaze brown decorative for the protection of Ming Dynasty underglaze brown decorative [10–12].

At present, traditional algorithms are widely used in image vision. In the analysis and processing of geometric and statistical features based on image pixels, traditional algorithms have developed many different applications [10, 13, 14]. Among them, the most common applications include image enhancement, image segmentation, image recognition and target tracking [15]. However, due to the complex pattern features of the Ming Dynasty underglaze brown decorative, the pattern extraction of the underglaze brown decorative cannot be obtained through simple image enhancement and image segmentation. It is necessary to design an algorithm suitable for the extraction of underglaze brown decorative pattern based on the actual situation [16, 17].

Based on the complexity of porcelain decorative patterns information, we have proposed a pattern extraction method that combines single scale gamma correction with grayscale sharpening method. Improve the contrast and brightness of the image by nonlinear transformation. Meanwhile, to solve the problem of fuzzy image information, construct gray sharpening to enhance the high-frequency component and improve the clarity of the image. The combination of the two can achieve a good image enhancement effect and improve the effect of image segmentation. This method is not only suitable for the pattern extraction of the underglaze brown decorative image, but also can achieve good results on other images, and has guiding significance for the enhancement and segmentation of other images.

## 2 Algorithm flow chart & Ming Dynasty underglaze brown decorative image feature analysis

### 2.1 Algorithm flow chart

**Fig 1:**
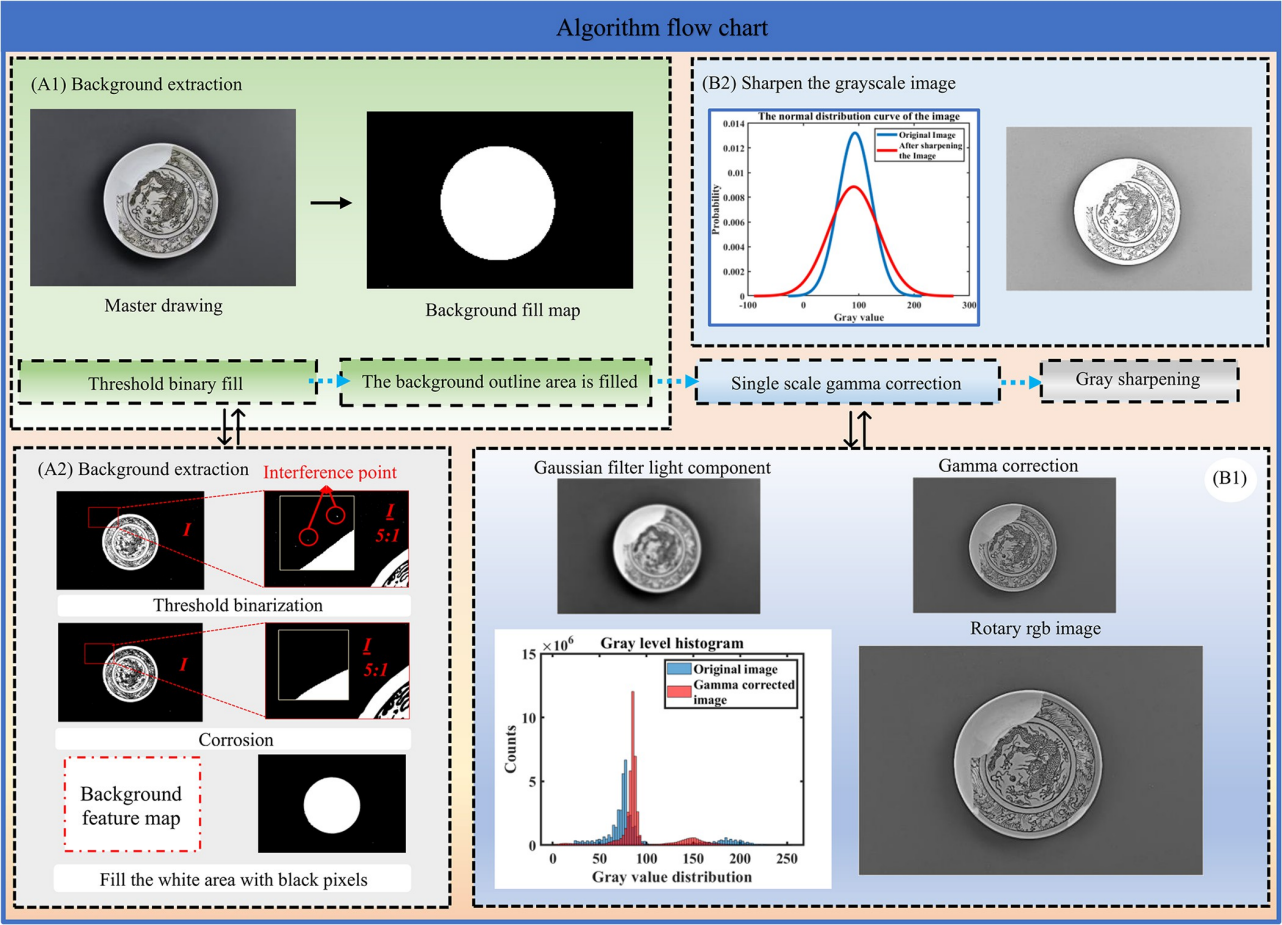
Algorithm flow chart of the extraction method of elemental underglaze brown decorative pattern based on the coupling of single-scale gamma correction and gray sharpening.

Ming Dynasty underglaze brown decorative is one of the classic representatives of Chinese traditional ceramic culture, and its unique patterns and colors have become the object of extensive collection and research in the world. However, due to the complexity of the pattern and the variability of the background, it is difficult for traditional image processing methods to extract the complete pattern of the pattern. Therefore, aiming at the problem of complete pattern extraction of primary underglaze brown decorative, this paper proposes a method of primary underglaze brown decorative pattern extraction based on the coupling of single-scale gamma correction and gray sharpening. As shown in Fig 1, the algorithm flow chart (1)-(2) of the extraction method of elemental underglaze brown decorative pattern based on the coupling of single-scale gamma correction and gray sharpening shows, the method includes three parts: background extraction, pattern enhancement and pattern segmentation.

Fig 1A1, 1A2 shows the background extraction module. In the background extraction module, it includes two steps, step 1 threshold binarization filling and background contour region filling. Firstly, the threshold function, which is more accurate than the general binarization function, is used for binarization, and the morphological operation function is used to fill in the small pattern information residue, and then the edge contour recognition and filling algorithm is constructed to recover the complete background information. Through this step, the background information can be effectively eliminated, laying a foundation for the subsequent pattern extraction.

Fig 1B1, 1B2 shows the pattern enhancement module. In the pattern enhancement module, single-scale gamma correction and gray sharpening methods are used to improve the contrast and clarity of the image. In the single scale gamma correction, the V channel of HSV image is selected, and the appropriate gamma value is selected for 2D gamma correction through Gaussian filtering, and then synthesized and converted into RGB image to make the brightness and shade of the primary underglaze brown decorative pattern more uniform. In gray sharpening, 3x3 convolution kernel is used for point by point average sharpening to enhance the edge information of the image. These steps can effectively reduce the noise in the image and improve the clarity of the image.

**Fig 2:**
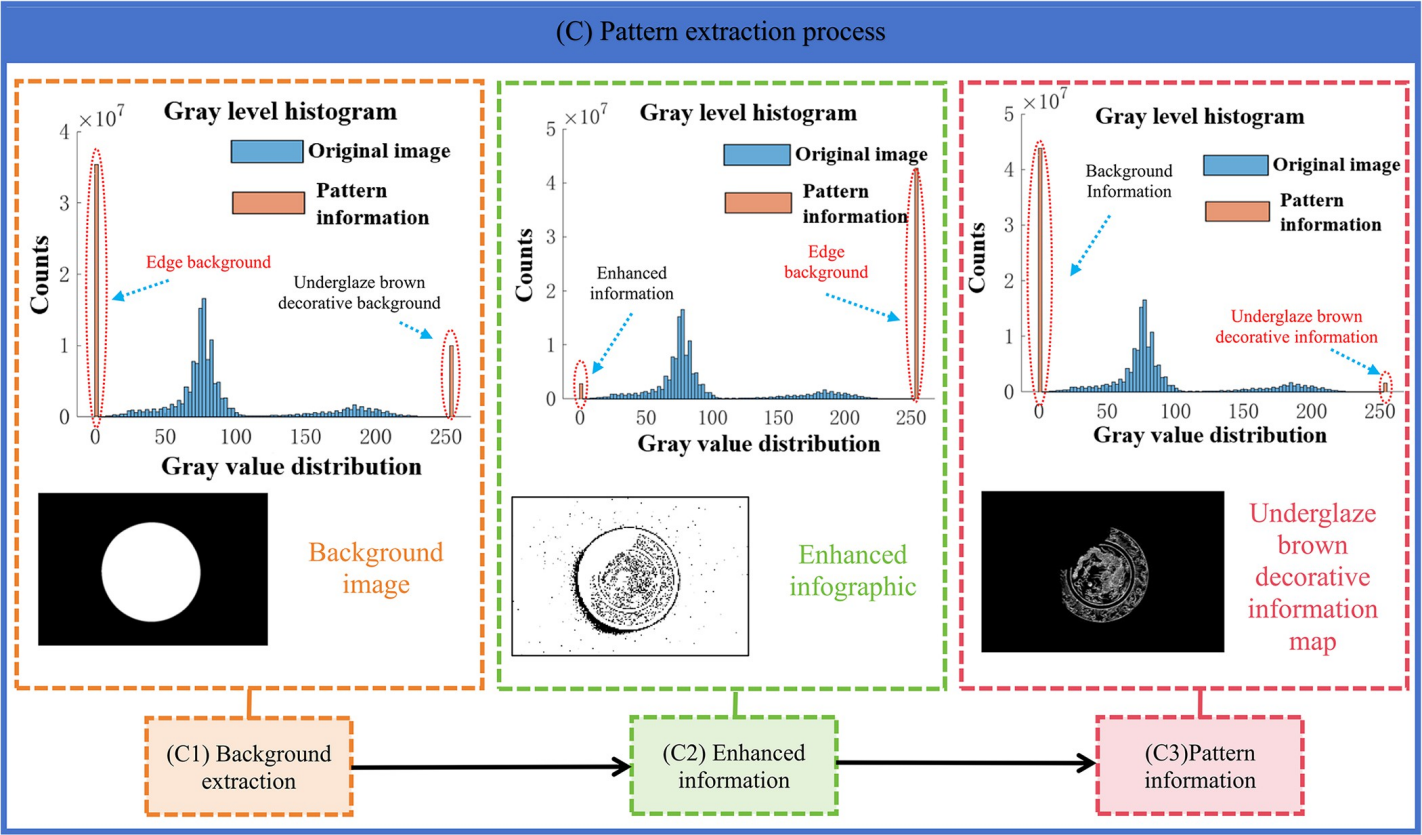
Algorithm flow chart of the extraction method of elemental underglaze brown decorative pattern based on the coupling of single-scale gamma correction and gray sharpening.

The Fig 2(C1–C3) shows the pattern extraction module. In the pattern extraction module, the optimized underglaze brown decorative pattern information obtained from the pattern enhancement and the background information obtained from the background extraction are separated from the background by matrix subtraction operation. This step needs to use the matrix operation technology in image processing, which can effectively separate the underglaze brown decorative pattern from the background and achieve the purpose of extracting the complete pattern. Finally, the correct information of the image is further recovered by the stretch excise operation.

### 2.2 Ming Dynasty underglaze brown decorative image feature analysis

**Fig 3:**
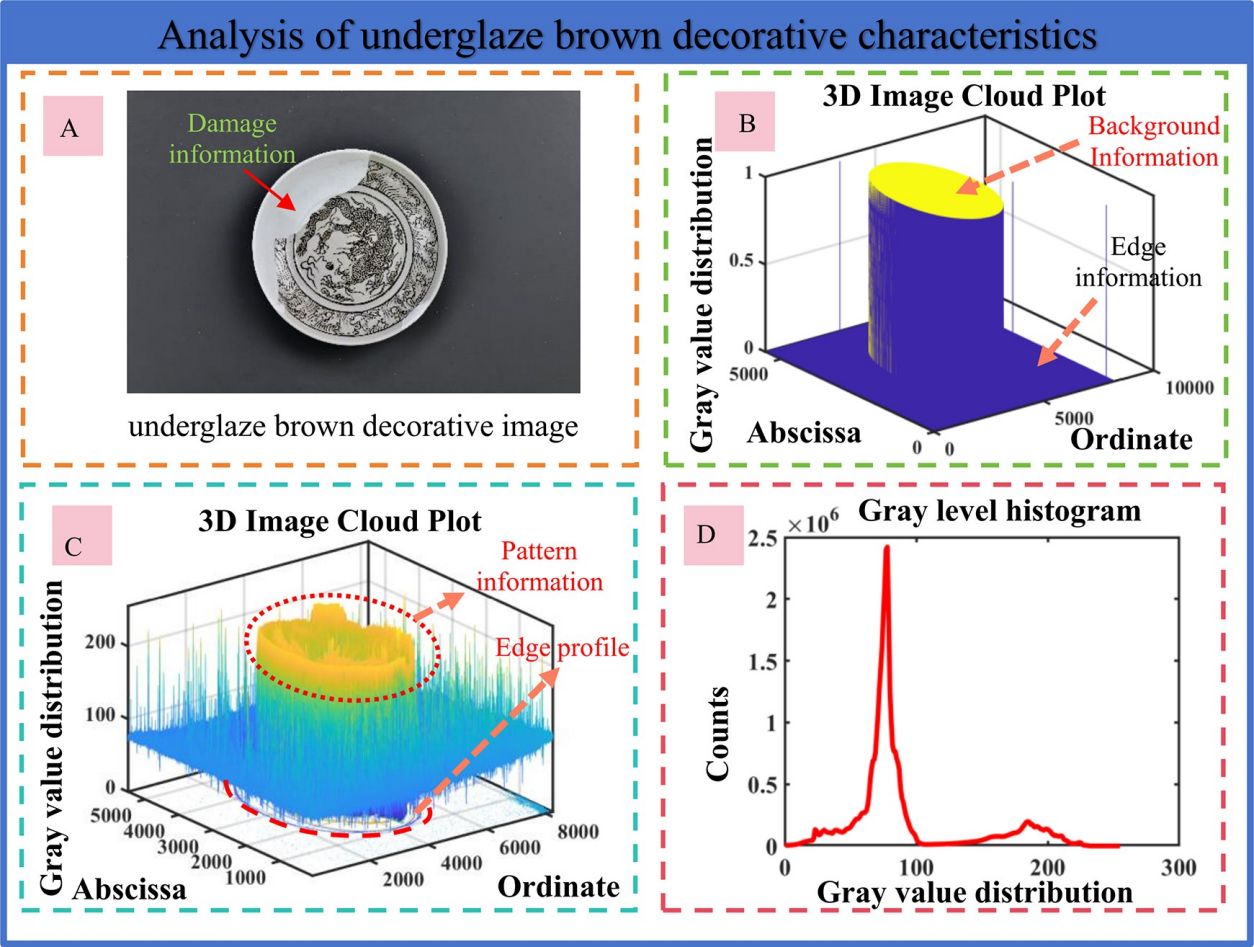
Ming Dynasty underglaze brown decorative feature analysis diagram. Firstly, Figure (A) shows an image of a underglaze brown decorative plate. It can be seen that the large white part of the decoration on the edge of the porcelain plate has fallen off, and there is a reflection on the porcelain surface of the image caused by the shooting. These problems will affect the quality of the image, so it is necessary to take the corresponding digital image processing technology to repair and enhance. Secondly, Figure (B) shows the background of the Ming Dynasty underglaze brown decorative plate. By analyzing the background features of the porcelain plate in the image, the patterns in the image can be better understood and interpreted. Third, Figure (C) shows the three-dimensional cloud image of the Ming Dynasty underglaze brown decorative plate, which is used to analyze the three-dimensional shape of the color value of the pattern and the edge outline of the porcelain plate background. This information is very helpful for understanding and interpreting the patterns and patterns of Ming Dynasty underglaze brown decorative plates. Finally, Figure (D) is the histogram of color values of the Ming Dynasty underglaze brown decorative plate. The Figure is used to analyze the degree of color concentration of the primary underglaze brown decorative image, and the most concentrated position is also the main concentration point of the pattern value. Through this Figure, we can better understand and interpret the color characteristics of the Ming Dynasty underglaze brown decorative plate.

The Ming Dynasty underglaze brown decorative plate is one of the traditional Chinese ceramic works of art, famous for its exquisite painting skills and rich cultural connotations in the Ming and Qing dynasties. However, due to the long history and different preservation conditions, there may be some problems in the digital image, such as the absence of reflective effect, image quality decline, etc., these problems will affect the accurate analysis and research of its artistic characteristics. In order to extract patterns better, this paper designs Fig 3. Ming Dynasty underglaze brown decorative feature analysis chart. As shown in the Figure, the graph is divided into four parts:

In order to improve the visual effect and feature interpretation ability of the underglaze brown decorative plate image, this paper designs a pattern enhancement module combined with the image enhancement technology in the field of digital image processing, which is used to solve the problems of image noise and reflection. By using these techniques, the quality and clarity of the image can be improved, so as to better analyze and interpret its artistic characteristics.

## 3 Background extraction algorithm based on single-scale gamma correction and gray sharpening

Image enhancement is an important branch in the field of image processing, which refers to the use of digital signal processing technology to make the image to achieve better sensory effect or to meet the specific application needs. One of the common image enhancement methods is based on histogram equalization, which can make the dynamic range of the image larger, enhance the contrast of the image, and make the image clearer. However, histogram equalization also has its limitations, for the image with uneven illumination, the effect of histogram equalization will become worse, and sometimes even lead to the loss of image information. Therefore, in order to deal with images with uneven illumination, a background extraction algorithm based on single-scale gamma correction and gray sharpening is constructed.

### (1) Single-scale gamma correction

**Fig 4:**
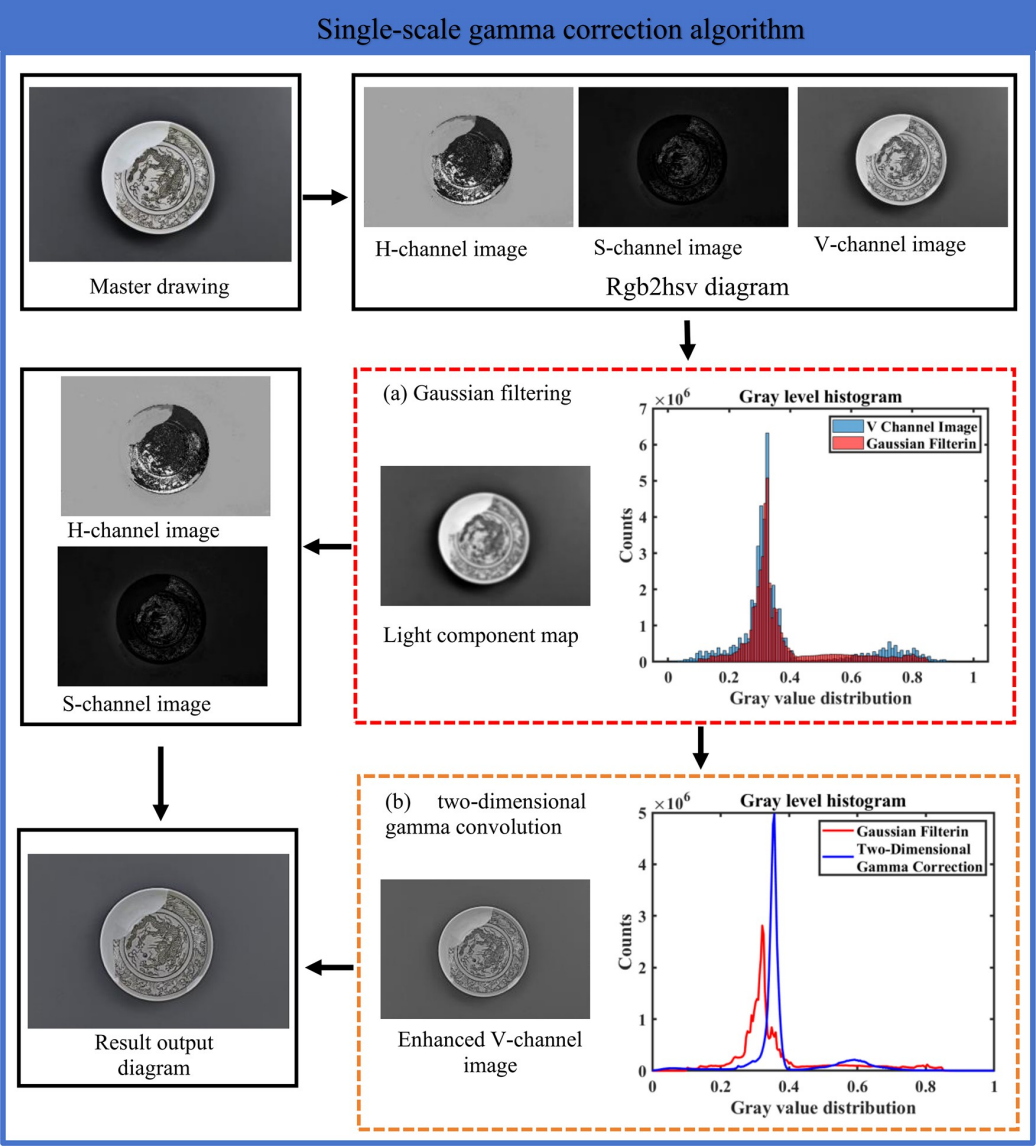
Flow chart of single-scale gamma correction algorithm.

Aiming at the problem of uneven illumination in the underglaze brown decorative images, a single-scale gamma correction function is constructed, as shown in Fig 4. The flow chart of averaging the brightness threshold of the two-color channel is shown. The algorithm first converts the input image into HSV space and processes the brightness component. Then, Gaussian filtering is used to remove the illumination inhomogeneity, and two-dimensional gamma convolution is used to correct the brightness. Finally, the processed HSV image is converted to RGB image output.

In this method, the input image is first converted from RGB color space to HSV color space, and the brightness channel is smoothed by Gaussian filtering. This step can be expressed by the following formula:

$$\begin{array}{l} h\_\{i,j\}=H/360;\\ s\_\{i,j\}=S/100;\\ v\_\{i,j\}=V/100. \end{array}$$ (1)

Where, H,S, and V are the brightness, saturation, and value of pixels (i,j) in the input image, respectively.

Next, as shown in Fig 4(A), Gaussian filter is constructed to smooth the brightness channel to reduce the influence of noise and illumination changes. The kernel of the Gaussian filter can be expressed by the following formula:

HSIZE=min(rows,cols) (2)

$$q=\sqrt{2},sigma=50$$ (3)

$$F_{i,j}=\frac{1}{2\pi \sigma^{2}}\cdot \exp(-\frac{{\left(i-\frac{HSIZE+1}{2}\right)}^{2}+{\left(j-\frac{HSIZE+1}{2}\right)}^{2}}{2\sigma^{2}})$$ (4)

HSIZE: The size of the Gaussian filter kernel, SIGMA: the standard deviation of the Gaussian filter, rows and cols are the number of rows and columns of the input image respectively, F is the Gaussian filter kernel, exp is the exponential function.

Then, as shown in Fig 4(B), two-dimensional gamma convolution is built to correct the image to improve the image quality under lighting conditions. According to the average brightness value m of the image, the following formula is used to correct the image:

$$m=\frac{1}{N}\sum_{i=1}^{N}g_{i}$$ (5)

$$gama_{i,j}=0.5^{(m-gaus_{i,j})/m}$$ (6)

$$out_{i,j}=v_{i,j}^{gama_{i,j}}$$ (7)

Where N is the total number of pixels in the input image, g is the image after Gaussian filtering, and out is the output image.

In this study, a single-scale gamma correction algorithm was used to solve the problem of uneven illumination in the underglaze brown decorative image. By converting the image to the HSV color space, the luminance channel was smoothed by Gaussian filtering, the gamma value in the paper was used, the size of the Gaussian convolution kernel was set to the minimum number of rows and columns of the input image, and finally the V channel was recombined with the H and S channels into the enhanced result output map. By adjusting and correcting the brightness, especially in the case of insufficient or excessive lighting, the final output image is enhanced to adapt to different lighting conditions, which improves the visual effect of the underglaze brown decorative image.

In this study, the size of the Gaussian convolution kernel is set to the minimum of the number of rows and columns in the input image using the gamma value in the paper. The final output image is enhanced, which can effectively improve the image quality, especially in the case of insufficient or excessive light. Finally, the V channel is re-combined with H and S channels to transform the enhanced output graph.

### (2) Grayscale sharpening algorithm

**Fig 5:**
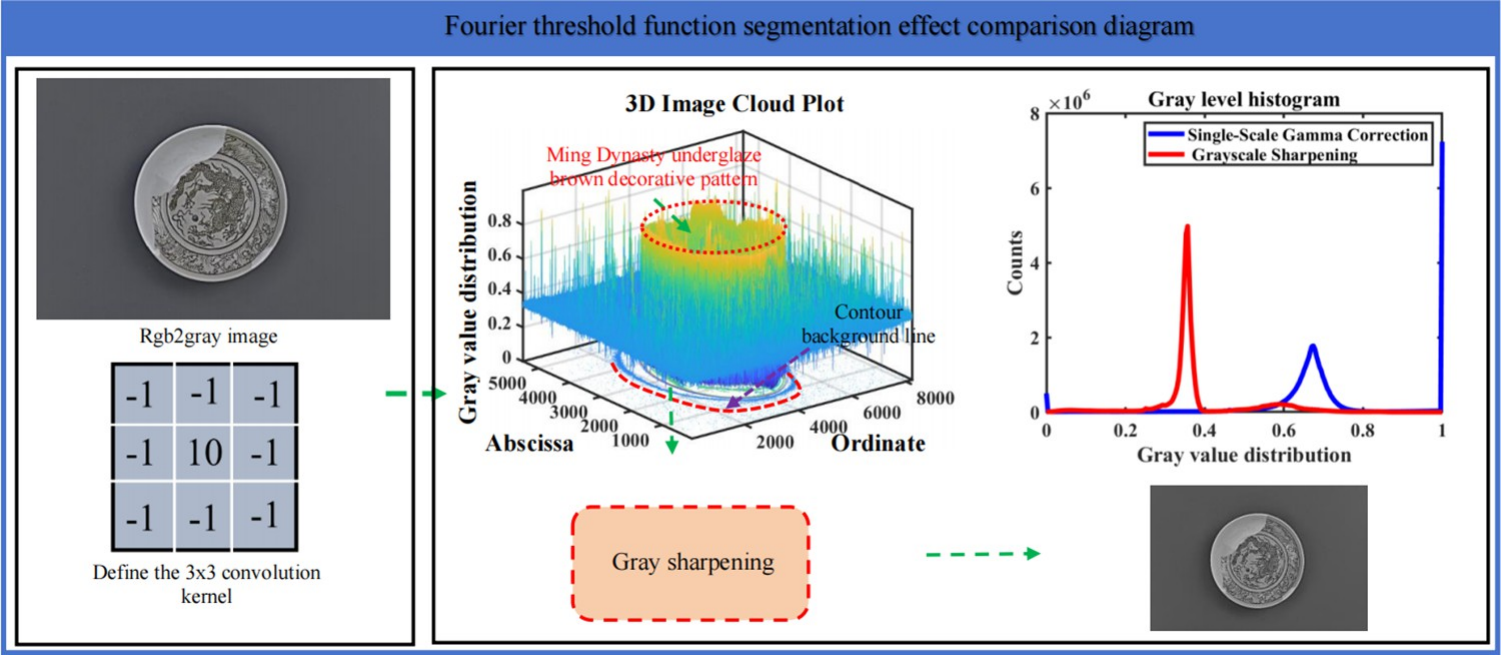
Comparison of Fourier threshold function segmentation effect.

In order to improve the clarity and detail of the image, we designed a 3x3 convolution kernel to sharpen the input image, as shown in Fig 5. Fourier threshold function segmentation effect comparison Figure is shown. The numerical coefficients in the convolution kernel play a crucial role in sharpening the image effect. In this algorithm, we define a sharpened convolution kernel, in which the center pixel has the highest weight and the surrounding pixel has the lower weight, so as to highlight the edges and details of the image.

With the convolution operation, we can replace the value of each pixel with the weighted average of its surrounding pixels, thus enhancing the edges and details of the image. This allows us to see the features in the image more clearly, making the image more realistic. The sharpened image can better present details, increasing the visual effect and artistry of the image.


$$K=[\begin{array}{l} -1-1-1\\ -1-10-1\\ -1-1-1 \end{array}]$$ (8)

Define the 3×3 convolution kernel k.

$$(I*K)i,j=\sum m\sum_{n}I_{m,n}K_{i-m,j-n}$$ (9)

Where *I* is the input image, K is the convolution kernel, and (I*K)(i,j) is the value of the pixel (i,j) in the output image.

It is worth noting that in the sharpening process, combined with the selection of the numerical coefficients of the convolution kernel and the relationship between image pixels, the coefficients of the convolution kernel can be changed according to the needs to improve the effect of image sharpening. Therefore, in practical application, we can select and adjust the convolution kernel according to the specific needs and image characteristics to achieve the best sharpening effect.

## 4 Result graph analysis

### (1) Demonstration of the enhancement effect of single-scale gamma correction and gray sharpening

**Fig 6:**
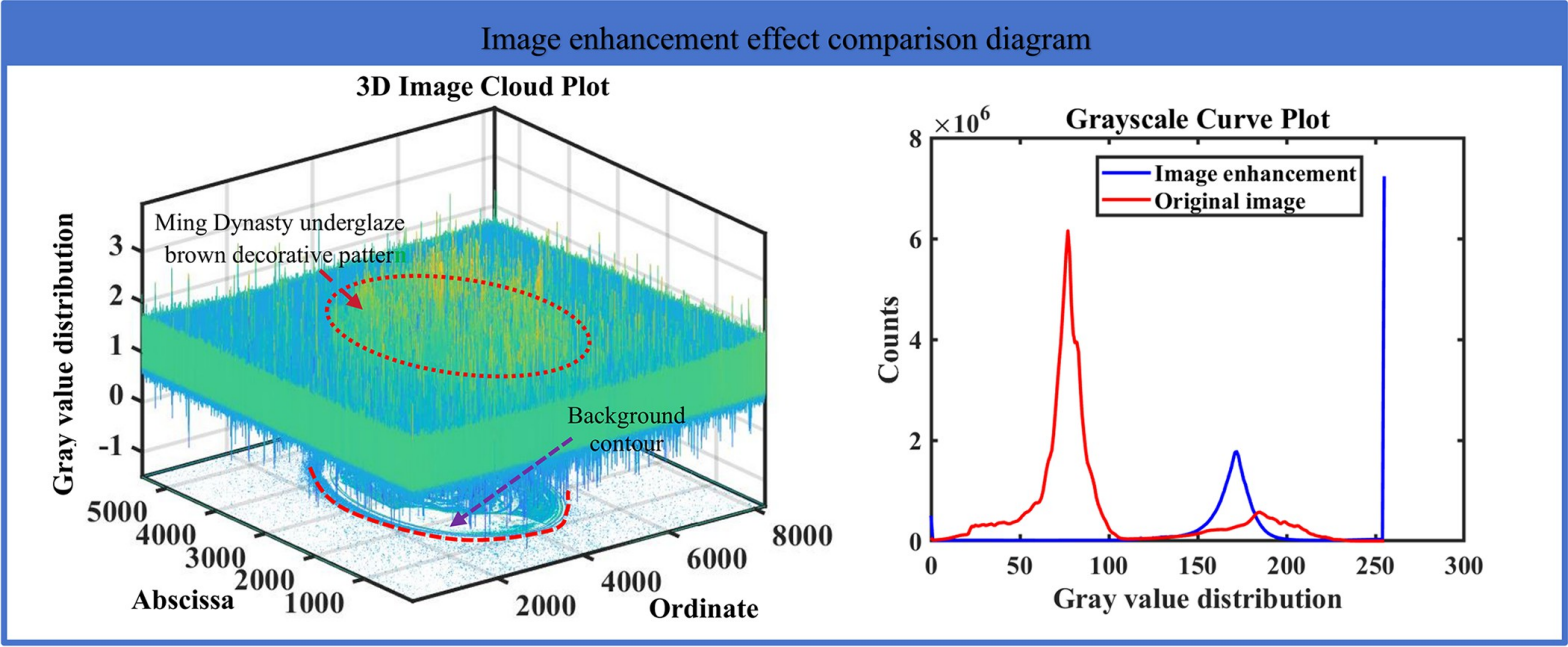
Comparison of the effect of single-scale gamma correction and gray sharpening.

In the image segmentation processing, image enhancement is an effective method to improve image quality, and combining image enhancement methods can improve the effect of image segmentation. Therefore, this paper combines single-scale gamma correction and gray sharpening methods to improve the effect of Ming Dynasty underglaze brown decorative segmentation. In order to show the effect of image enhancement in this paper, a comparison diagram of the effect of single-scale gamma correction and gray sharpening is constructed in Fig 6 and Table 1. The comparison table of image enhancement effect of single scale gamma correction algorithm is used to show the image enhancement effect.

**Table 1:** Comparison of image enhancement effect of single-scale gamma correction algorithm.

	PSNR(dB)	RMSE	The information entropy of the original image	The enhanced information entropy value
Histogram equalization	13.011466	57.012078	6.549082	5.505031
Contrast stretching	17.319919	34.717164	6.549082	**5.964700**
Single scale gamma correction	**22.618605**	**18.862964**	6.549082	5.800973

Fig 6 construct an image enhancement method. The image realizes the preliminary classification of pattern information through stretching and other methods, and extends the fuzzy information of the image into the color value region. As shown in the right Figure, the color value information of the original image is expanded from the original concentrated region to the relatively dispersed color value region of the image enhancement through the image enhancement method. As shown in Table 1, four evaluation indexes, including peak signal-to-noise ratio (PSNR), structural similarity index (SSIM), root mean square error (RMSE) and information entropy, are used to analyze the image enhancement effects of the proposed methods.

Peak signal-to-noise ratio (PSNR) is a common index to measure the similarity between two images, and its calculation formula is as follows:

$$PSNR=10\times log10(255^{2}/MSE)$$ (10)

Where MSE is the mean square error, defined as the average sum of the squares of the difference between the values of each pixel between two images. The higher the value of PSNR, the higher the similarity between the two images.

The root mean square error (RMSE) is a measure of the difference in pixel values between two images and is calculated as follows:

$$RMSE=sqrt(mean((img1-img2).^{2}))$$ (11)

Among them, img1 and img2 represent two images respectively, mean means to find the average value, sqrt means to find the square root. The smaller the value of RMSE, the smaller the difference in pixel values between the two images.

Information entropy is an index to measure the amount of image information, and its calculation formula is as follows:

H=−sum(p.*log2(p)) (12)

Where p is the histogram of the image, log2 represents the logarithm base 2, and sum represents the sum. The higher the value of information entropy, the greater the amount of information contained in the image.

In this paper, the above four evaluation indicators are constructed to evaluate the image. By calculating PSNR, SSIM and RMSE, the enhancement effect and quality of image processing algorithms are evaluated. By calculating the information entropy, the information contained in the image can be evaluated, and the performance and effect of the image processing algorithm can be evaluated comprehensively. After comparison, it can be found that the multi-scale gamma correction on PSNR and RMSE has the best effect, and the information entropy ranks second, which is 22.618605, 18.862964 and 5.800973, respectively.

### (2) Ming Dynasty underglaze brown decorative segmentation effect display

**Fig 7:**
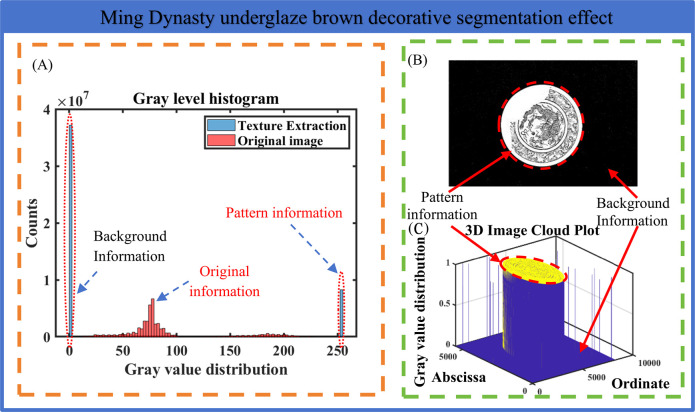
Analysis of the effect of underglaze brown decorative segmentation. As shown in Figure (A), the basic underglaze brown decorative pattern extraction method based on the coupling of single-scale gamma correction and gray sharpening is divided into binary information of 0–1 from the primary underglaze brown decorative images distributed in 0-255RGB image color values. The pattern information as shown in Figure (B) is successfully segmented, and the porcelain plate background and edge information of the Ming Dynasty underglaze brown decorative image are segmented. As shown in Figure (C), the pattern information is a graph composed of 1 color block on the horizontal and vertical coordinates of the image.

For the display of the extraction effect of the underglaze brown decorative pattern, the analysis diagram of the segmentation effect of the underglaze brown decorative pattern is constructed in Fig 7, which is used to display the segmentation effect of the underglaze brown decorative pattern, including gray histogram, segmentation map and three-dimensional cloud image.

The method proposed in this paper successfully extracts the pattern information of the Ming Dynasty underglaze brown decorative from the image information, and achieves good segmentation accuracy and effect.

### (3) Comparison of segmentation effect between different algorithms

**Fig 8:**
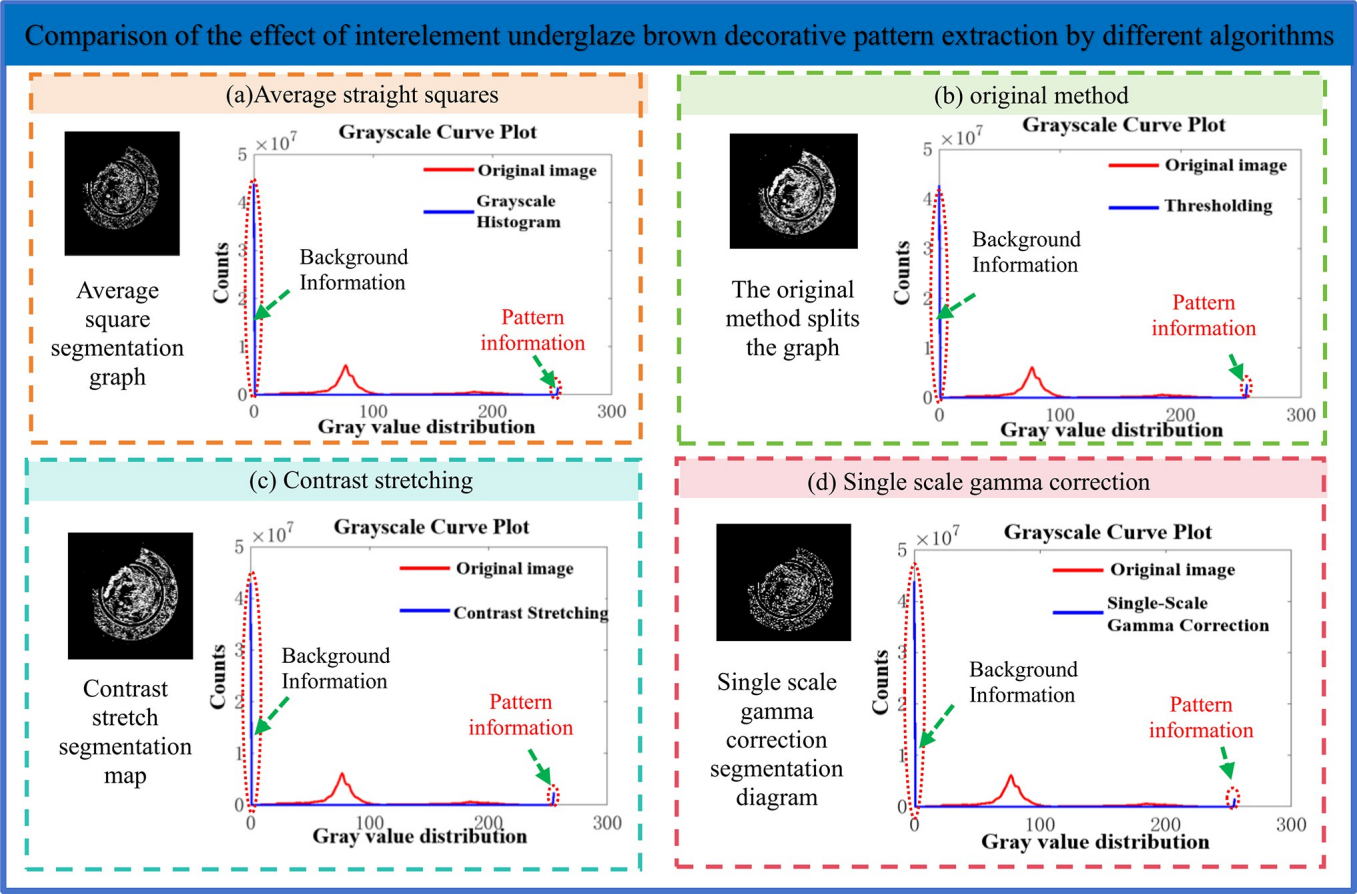
Comparison of segmentation effect between different algorithms.

In view of the effect display of the extraction of underglaze brown decorative pattern, the comparison diagram of segmentation effect between different algorithms and Table 2 are constructed in Fig 8. The comparison table of the effect index of the enhanced method’s underglaze brown decorative segmentation is used to compare the effect of the pattern extraction method in this paper. Fig 8 includes the average straight square, the original method, contrast stretching and the single-scale gamma correction segmentation method in this paper.

**Table 2:** Comparison table of the effect indicators of the enhanced methods.

Method	Evaluation index				
	F1Score	Accuracy(%)	Miou(%)	Recall	Precision
Original method	0.88298	**98.8754**	77.243	0.78637	0.84021
Average straight method	0.90449	92.456	78.026	0.90538	0.89103
Contrast stretching	0.86224	97.452	78.354	0.78694	0.86237
This text method	**0.92745**	92.745	**82.253**	**0.97942**	**0.92458**

According to the comparison between the segmentation effect of different algorithms in Fig 8A–8D, the four methods have successfully segmented the underglaze brown decorative pattern from the Ming Dynasty underglaze brown decorative image, and divided the Ming Dynasty underglaze brown decorative image into background information and pattern information. Compared with the pattern information, the pixel segmentation of the original method and the contrast stretching method has high accuracy, but there are a lot of segmentation errors, and the average crossover ratio is not good. The average square method has low error rate but low pixel accuracy. The single scale gamma correction is between the three, which improves the segmentation accuracy and achieves good segmentation effect under the premise of ensuring the accuracy rate.

As shown in Table 2. Comparison table of the effect indicators of the segmentation effect of the enhanced methods, the proposed extraction method based on the coupling of single-scale gamma correction and gray sharpening achieves the best results in F1Score, Miou(%), Recall and Precision, ranking third in Accuracy(%). They were 92.745, 82.253, 0.97942, 0.92458 and 0.92745, respectively.

### (4) Ablation experiment

**Fig 9:**
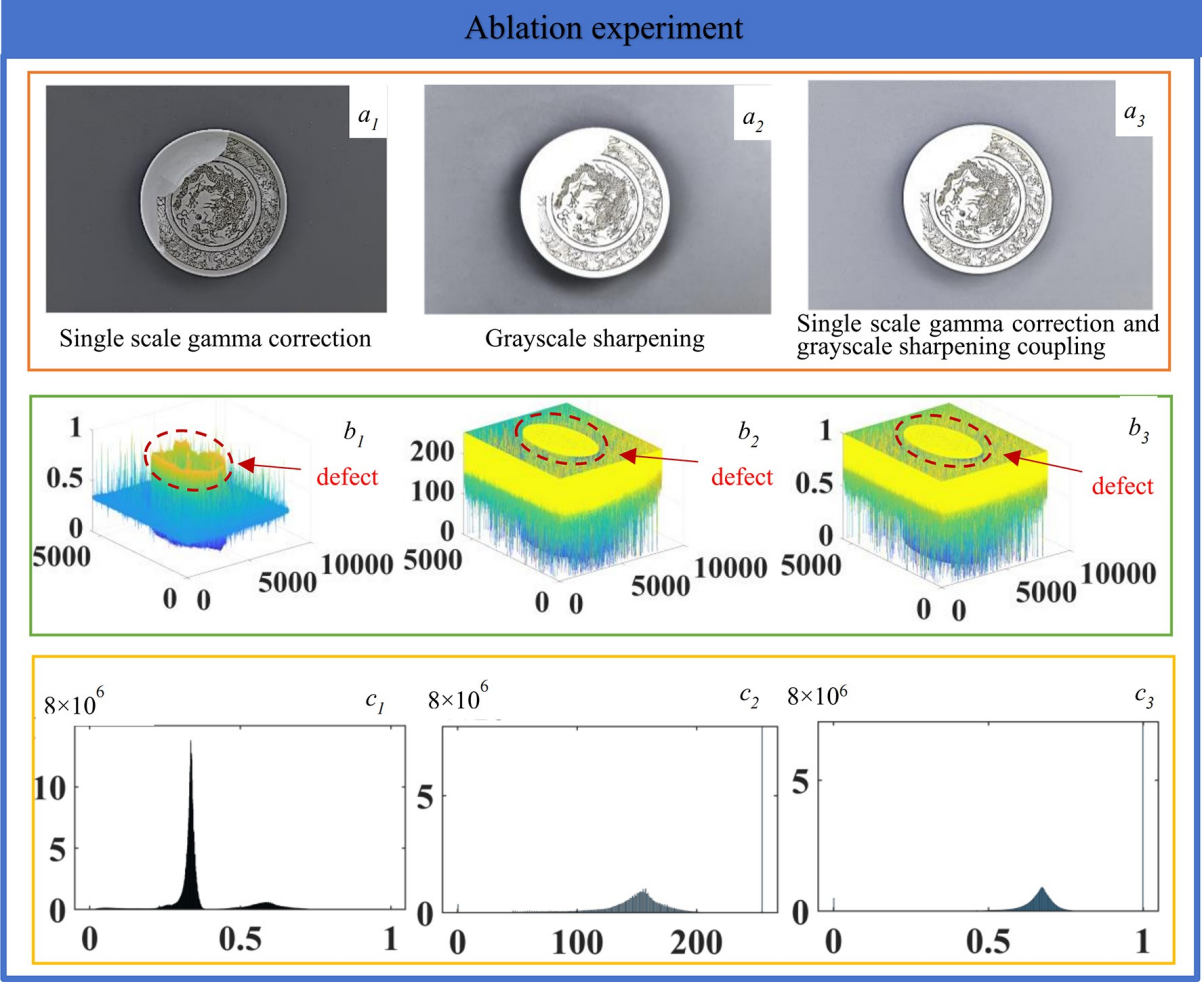
Ablation experiment.

The single-scale gamma correction is used to enhance the brown decorative pattern as shown in Fig 9*A*_*1*_, the overall brightness of the image is low, eliminates the phenomenon of uneven image lighting, but the details at the edge of the pattern are missing. The three-dimensional cloud map of the gray value is shown in Fig 9*B*_*1*_, strengthens the pattern information, and the pattern information is clear and the background information is complete. The histogram is shown in Fig 9*C*_*1*_, the gray values are not evenly distributed, reduces the brightness of the image and removes the reflection of the image. As shown in Fig 9*A*_*2*_, the pattern enhancement effect of the grayscale sharpening algorithm strengthens the edges of the image and improves the clarity of the pattern, but the background lighting is dark and the lighting is uneven. The grayscale value of the three-dimensional cloud is shown in Fig 9*B*_*2*_, and the pattern information and the background information are distinct, strengthens the edge detail information of the image. The histogram is shown in Fig 9*C*_*2*_, and the gray values are mainly concentrated in the values between 100 and 200 and 255, and the background information is clearer. As shown in Fig 9*A*_*3*_, the image illumination is evenly distributed, making the edges more prominent and improving the clarity of the image. The 3D contour of grayscale values is shown in Fig 9*B*_*3*_, enhances the edge details of the image and improves contrast and clarity. The histogram is shown in Fig 9*C*_*3*_, the gray values are centrally distributed between 0.5 and 1, as well as on the values 0 and 1, and the pattern information and background information are distinct. The three enhancement algorithms have a certain effect on the information enhancement of the underglaze brown decorative pattern, among the coupling enhancement algorithm of single-scale gamma correction and grayscale sharpening can adjust the brightness and contrast of the image, enhance the pattern information, make the image look brighter or darker as a whole, enhance the details and edges of the image, and make the image sharper.

**Fig 10:**
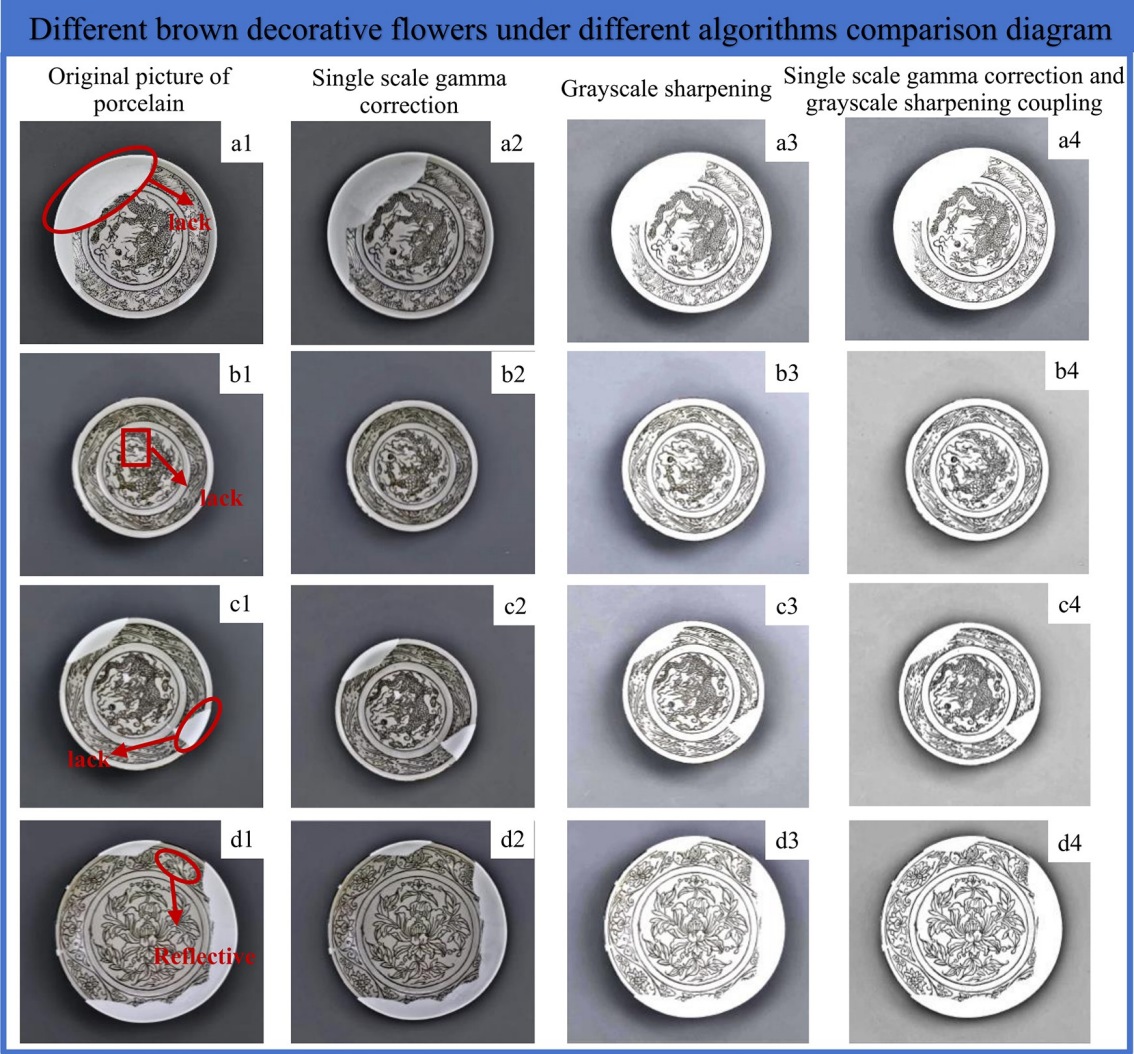
Comparison of different brown decorative flowers under different algorithms.

As shown in Fig 10*A*_*1*_–10*D*_*1*_, the original images of different underglaze brown decorative are characterized by the lack of pattern information and the phenomenon of surface reflection, and the pattern and the background cannot be effectively distinguished. The image obtained by single-scale gamma correction is the Fig 10*A*_*2*_–10*D*_*2*_, and the gray value of the image is adjusted to make the brightness and darkness of the underglaze brown decorative pattern more uniform. The Fig 10*A*_*3*_–10*D*_*3*_ is obtained under the effect of grayscale sharpening, and the convolutional kernel is used to sharpen the point by point to enhance the contrast of the edge of the image details. The single-scale gamma correction and grayscale sharpening are coupled to obtain the Fig 10*A*_*4*_–10*D*_*4*_, which reduces the noise of the underglaze brown decorative images and enhances the visibility of the underglaze brown decorative features.

In this paper, two evaluation indexes are constructed to evaluate the image enhancement effect of different brown decorative elements. The comparison of different algorithms is shown in Table 3.

**Table 3:** Comparison of enhancement indicators of different elements under different algorithms.

Brown decorative image	Experimental standard	Single scale gamma correction	Gray sharpening	Single scale gamma correction and gray-sharpening coupling
a1	SSIM	0.9285	0.8769	0.9792
	PSNR(dB)	22.618605	20.548921	23.867523
b1	SSIM	0.9782	0.8965	0.9927
	PSNR(dB)	29.781961	26.369852	30.059842
c1	SSIM	0.9619	0.7811	0.9919
	PSNR(dB)	28.517699	25.698572	29.726891
d1	SSIM	0.9142	0.7908	0.9741
	PSNR(dB)	23.738269	19.997563	24.098524

Structural similarity (SSIM) is a measure of the similarity between two images, and the peak signal-to-noise ratio (PSNR) is a measure of image quality. By calculating SSIM and PSNR, and evaluating the enhancement effect and quality of the image by the algorithm, different brown decorative elements are enhanced by different algorithms, and the coupling effect of single-scale gamma correction and grayscale sharpening is the best on SSIM and PSNR, the best SSIM and PSNR are b1, they are 0.9927 and 30.059842.

## 5 Conclusion

In this paper, the application of gamma correction and gray sharpening in the segmentation of Ming Dynasty underglaze brown decorative pattern is studied, and a method of elemental underglaze brown decorative pattern extraction based on the coupling of single scale gamma correction and gray sharpening is proposed. Through experimental verification, we found that both methods can effectively enhance the contrast, brightness and sharpness of the image, while preserving the details and texture information of the image. Gamma correction technology can adjust the brightness and contrast of the image, making the image brighter and clearer, while gray sharpening technology can enhance the details and texture of the image, making the image more rich and detailed, and the image enhancement reaches 22.618605, 18.862964 and 5.800973 in PSNR, SSIM and RMSE, respectively.

The pattern extraction method proposed in this paper and the two image enhancement methods combined have the advantages of fast calculation speed and good effect in practical application, and can be widely used in the fields of image enhancement, image processing and image analysis. The segmentation results of F1Score, Miou(%), Recall, Precision and Accuracy(%) reached 0.92745, 0.82253, 0.97942, 0.92458 and 0.92745, respectively. Finally, this study provides a simple and effective method to enhance image quality in the field of image processing, and provides a reference for the subsequent research and application.

## Supporting information

S1 Raw images Original picture. (ZIP)

S1 File Data indicators. (DOCX)

S2 File Source code. (DOCX)
