# Supplementary figures and images for: A new extraction method of underglaze brown decorative pattern based on the coupling of single scale gamma correction and gray sharpening
Source: PLoS One. 2024 Aug 29;19(8):e0305118. doi: 10.1371/journal.pone.0305118 (PMC11361591; doi:10.1371/journal.pone.0305118)

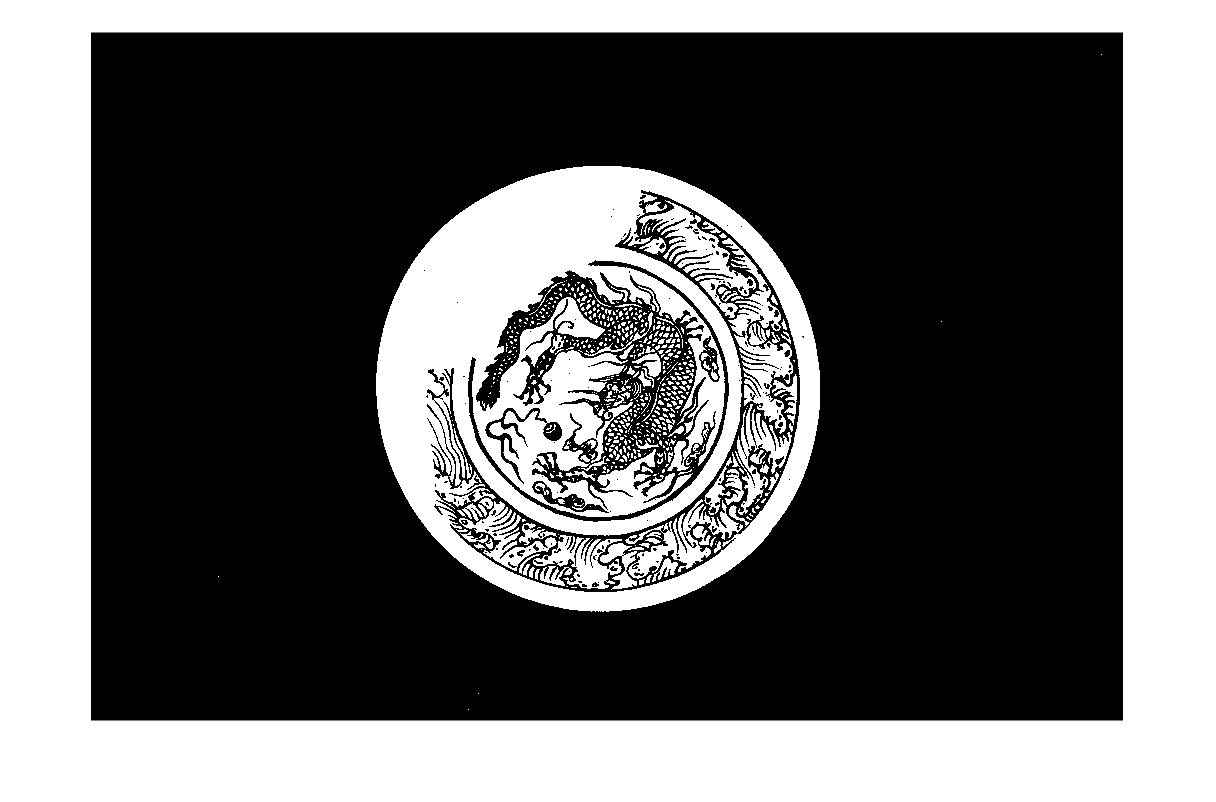

Supplement: S1 Raw images — (ZIP) [file pone.0305118.s001.zip › After corrosion.png]

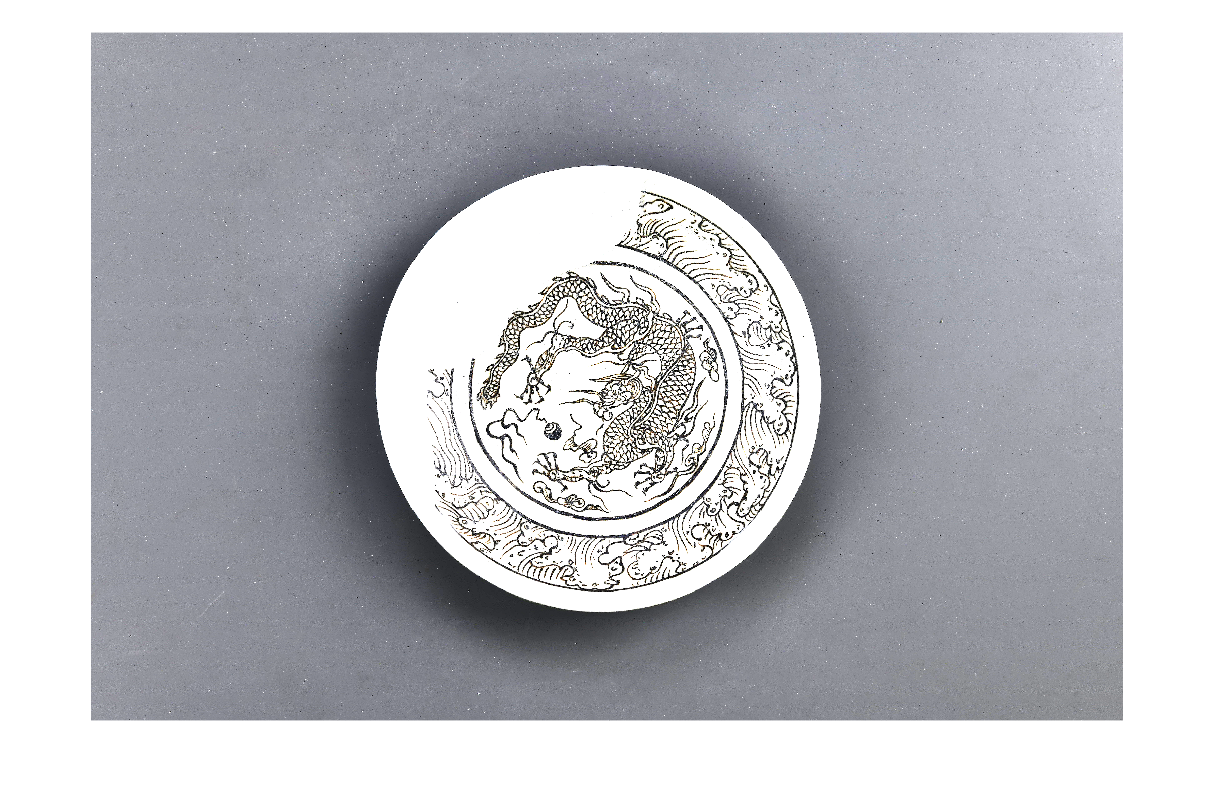

Supplement: S1 Raw images — (ZIP) [file pone.0305118.s001.zip › After sharpening.png]

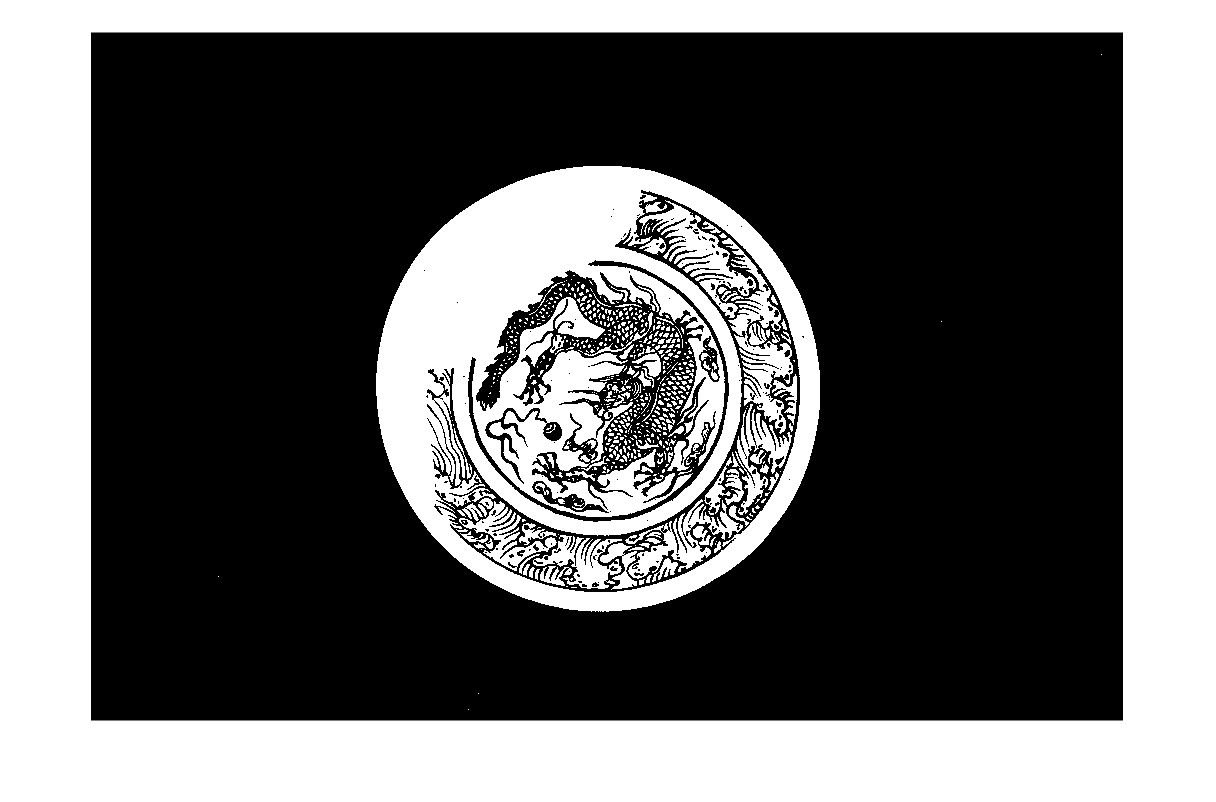

Supplement: S1 Raw images — (ZIP) [file pone.0305118.s001.zip › binaryzation .png]

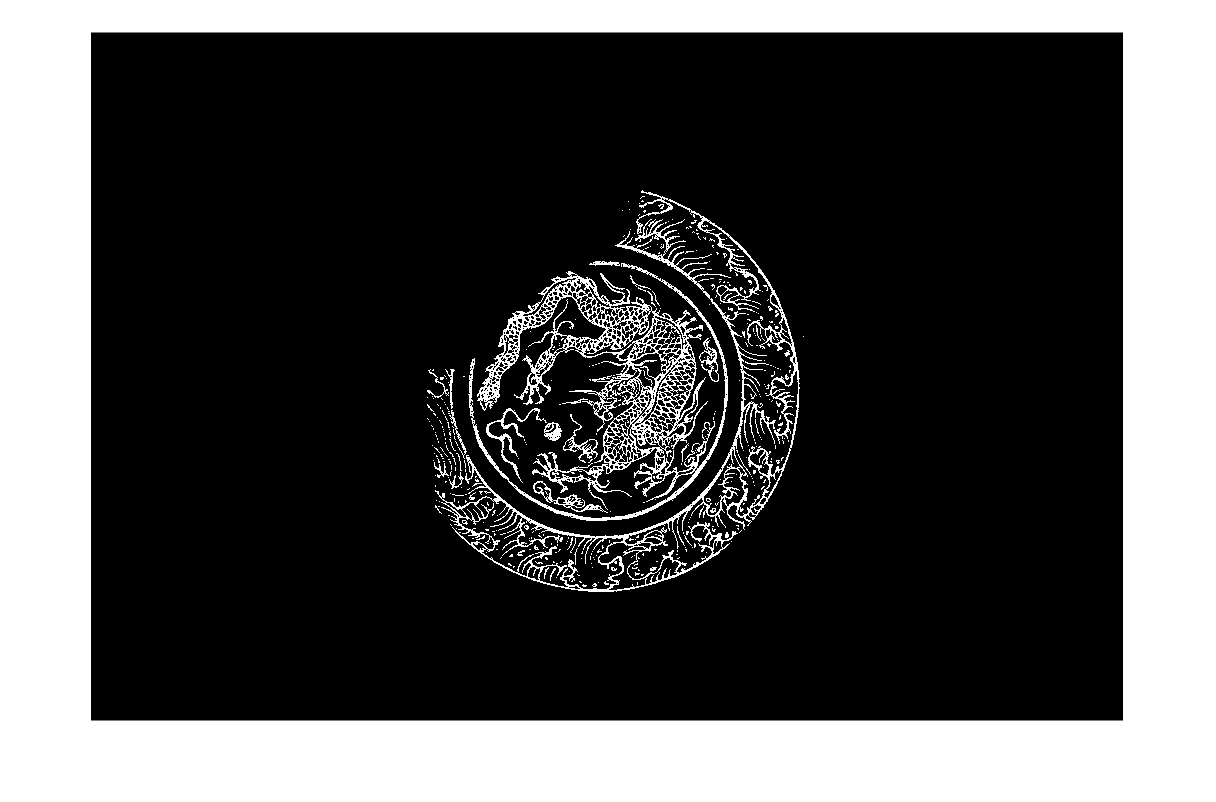

Supplement: S1 Raw images — (ZIP) [file pone.0305118.s001.zip › Final effect diagram after the matrix operation.png]

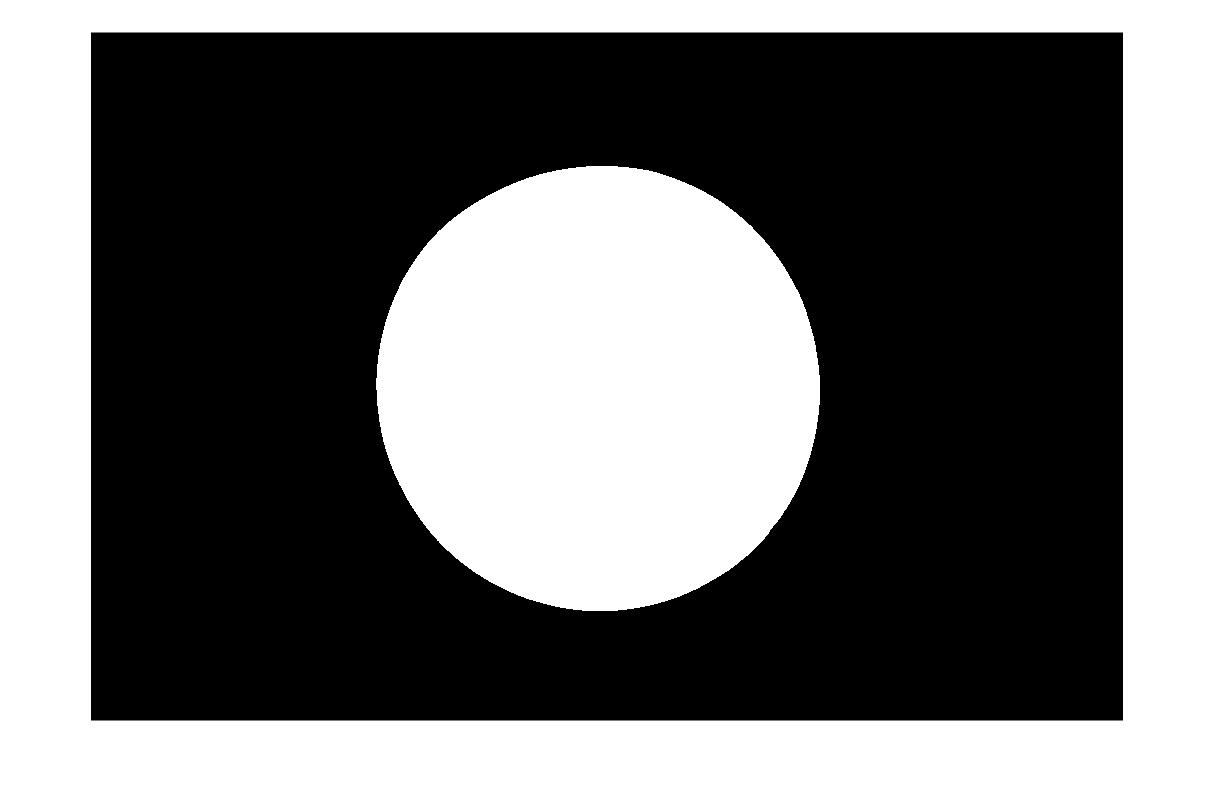

Supplement: S1 Raw images — (ZIP) [file pone.0305118.s001.zip › holes padding.png]

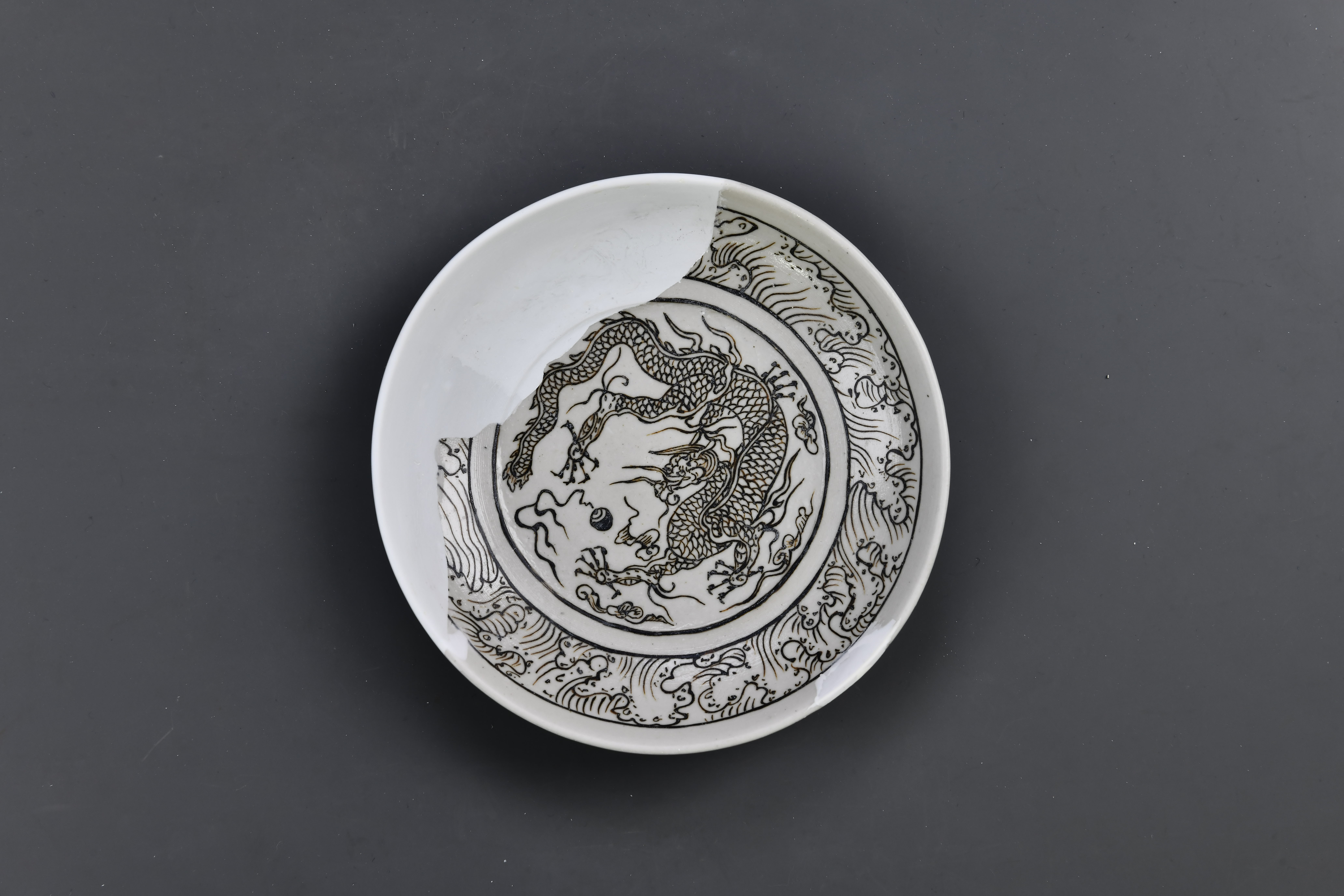

Supplement: S1 Raw images — (ZIP) [file pone.0305118.s001.zip › original drawing.JPG]

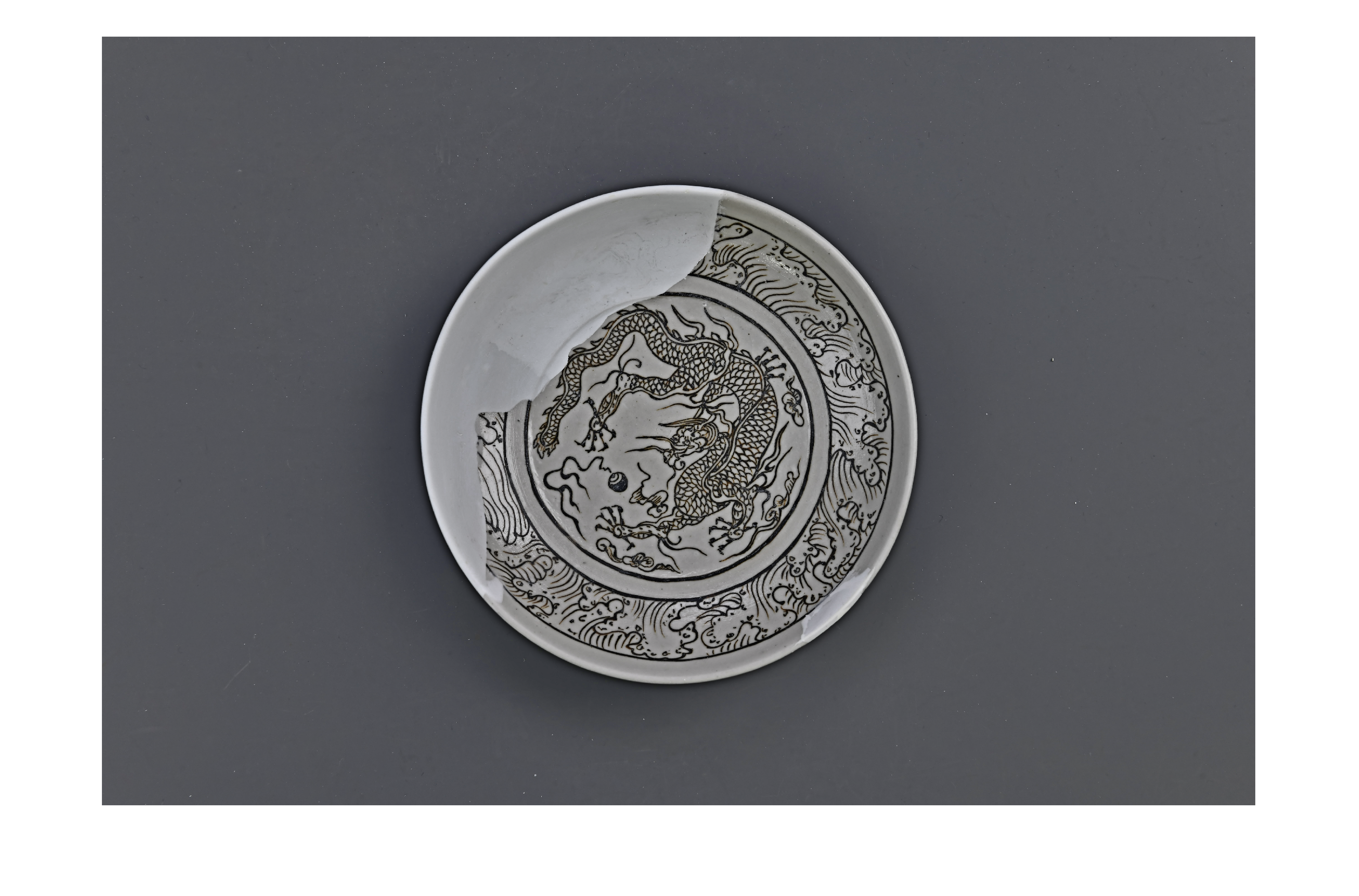

Supplement: S1 Raw images — (ZIP) [file pone.0305118.s001.zip › Single-scale gama correction.png]
