# Supplementary material for: A new extraction method of underglaze brown decorative pattern based on the coupling of single scale gamma correction and gray sharpening
Source: PLoS One. 2024 Aug 29;19(8):e0305118. doi: 10.1371/journal.pone.0305118 (PMC11361591; doi:10.1371/journal.pone.0305118)
Supplement: S1 File — (DOCX) [file pone.0305118.s002.docx]

Tab.1 Comparison of image enhancement effect of single-scale gamma correction algorithm

|  | **PSNR(**dB**)** | **RMSE** | **The information entropy of the original image** | **The enhanced information entropy value** |
| --- | --- | --- | --- | --- |
| Histogram equalization | 13.011466 | 57.012078 | 6.549082 | 5.505031 |
| Contrast stretching | 17.319919 | 34.717164 | 6.549082 | **5.964700** |
| Single scale gamma correction | **22.618605** | **18.862964** | 6.549082 | 5.800973 |

Tab.2 Comparison table of the effect indicators of the enhanced methods

| Method | Evaluation index | | | | |
| --- | --- | --- | --- | --- | --- |
|  | F1Score | Accuracy(%) | Miou(%) | Recall | Precision |
| Original method | 0.88298 | **98.8754** | 77.243 | 0.78637 | 0.84021 |
| Average straight method | 0.90449 | 92.456 | 78.026 | 0.90538 | 0.89103 |
| Contrast stretching | 0.86224 | 97.452 | 78.354 | 0.78694 | 0.86237 |
| This text method | **0.92745** | 0.92745 | **0.82253** | **0.97942** | **0.92458** |

Tab3. Comparison of enhancement indicators of different elements under different algorithms

| Brown decorative image | Experimental standard | Single scale gamma correction | Gray sharpening | Single scale gamma correction and gray-sharpening coupling |
| --- | --- | --- | --- | --- |
| a1 | SSIM | 0.9285 | 0.8769 | **0.9792** |
|  | PSNR(Db) | 22.618605 | 20.548921 | **23.867523** |
| b1 | SSIM | 0.9782 | 0.8965 | **0.9927** |
|  | PSNR(Db) | 29.781961 | 26.369852 | **30.059842** |
| c1 | SSIM | 0.9619 | 0.7811 | **0.9919** |
|  | PSNR(Db) | 28.517699 | 25.698572 | **29.726891** |
| d1 | SSIM | 0.9142 | 0.7908 | **0.9741** |
|  | PSNR(Db) | 23.738269 | 19.997563 | **24.098524** |
