# Supplementary material for: A new extraction method of underglaze brown decorative pattern based on the coupling of single scale gamma correction and gray sharpening
Source: PLoS One. 2024 Aug 29;19(8):e0305118. doi: 10.1371/journal.pone.0305118 (PMC11361591; doi:10.1371/journal.pone.0305118)
Supplement: S2 File — (DOCX) [file pone.0305118.s003.docx]

img = imread("C:\Users\admin\Desktop\underglaze brown decorative Pattern\G4：38.JPG");% Read in images and converted to grayscale images

grayImg = rgb2gray(img);

% Image was binarized using a threshold processing function

binaryImg = imbinarize(grayImg);

imshow(binaryImg)

% Binarized images were processed using morphological operator functions to fill the white area and remove small black objects

se = strel('square', 4);

% Conduct a corrosion operation on the image

binaryImg = imerode(binaryImg, se);

imshow(binaryImg)

filledImg = imfill(binaryImg, 'holes');

se = strel('disk', 3);

openedImg = imopen(filledImg, se);

imshow(openedImg);

im2=openedImg;

im=imread("C:\Users\admin\Desktop\underglaze brown decorative Pattern\G4：38.JPG");

[h,s,v]=rgb2hsv(im); % Go to the hsv space, for the brightness h treatment

% Gaussian filtering

imshow(v)

HSIZE= min(size(im,1),size(im,2));%The Gaussian convolution kernel size

q=sqrt(2);

SIGMA=50;%The c in the paper

F = fspecial('gaussian',HSIZE,SIGMA/q);

gaus= imfilter(v, F, 'replicate');

% gaus=(gaus*255);

figure;

imshow(gaus,[]);

title('Light component');

%2 D gamma convolution

m=mean(gaus(:));

[w,height]=size(v);

out=zeros(size(v));

gama=power(0.5,((m-gaus)/m));%Treatment according to the gamma correction formula

out=(power(v,gama));

figure;

imshow(out,[]);

rgb=hsv2rgb(h,s,out); %Turn back to the rgb space for display

imshow(rgb);

y=rgb2gray(rgb);

imshow(img)

im = y; % Read the grayscale image

imshow(y)

% image sharpening

kernel = [-1 -1 -1; -1 10 -1; -1 -1 -1]; % Defining convolutional kernels

im_sharp = imfilter(im, kernel); % Image sharpening was performed using the convolution kernel

figure, imshow(im_sharp), title('Images after sharpening');

f=im2bw(im_sharp,0.85);

imshow(f);

v=im2-f;

v=im2bw(v);

imshow(v)
